# Investigating a Machine Learning Approach to Predicting White Pixel Defects in Wafers—A Case Study of Wafer Fabrication Plant F

**DOI:** 10.3390/s24103144

**Published:** 2024-05-15

**Authors:** Dong-Her Shih, Cheng-Yu Yang, Ting-Wei Wu, Ming-Hung Shih

**Affiliations:** 1 Department of Information Management, National Yunlin University of Science and Technology, Douliu 64002, Taiwan; shihdh@yuntech.edu.tw; 2 Formosa Sumco Technology Corporation, Yunlin County 638501, Taiwan; roy@fstech.com.tw; 3 Department of Electrical and Computer Engineering, Iowa State University, 2520 Osborn Drive, Ames, IA 50011, USA; mshih@iastate.edu

**Keywords:** CMOS image sensor, white pixel defect, machine learning, wafer, prediction

## Abstract

CMOS image sensor (CIS) semiconductor products are integral to mobile phones and photographic devices, necessitating ongoing enhancements in efficiency and quality for superior photographic outcomes. The presence of white pixels serves as a crucial metric for assessing CIS product performance, primarily arising from metal impurity contamination during the wafer production process or from defects introduced by the grinding blade process. While immediately addressing metal impurity contamination during production presents challenges, refining the handling of defects attributed to grinding blade processing can notably mitigate white pixel issues in CIS products. This study zeroes in on silicon wafer manufacturers in Taiwan, analyzing white pixel defects reported by customers and leveraging machine learning to pinpoint and predict key factors leading to white pixel defects from grinding blade operations. Such pioneering practical studies are rare. The findings reveal that the classification and regression tree (CART) and random forest (RF) models deliver the most accurate predictions (95.18%) of white pixel defects caused by grinding blade operations in a default parameter setting. The analysis further elucidates critical factors like grinding load and torque, vital for the genesis of white pixel defects. The insights garnered from this study aim to arm operators with proactive measures to diminish the potential for customer complaints.

## 1. Introduction

Silicon wafers are fundamental in semiconductor manufacturing, undergoing multiple processes to become semiconductor components [1]. The industry’s growth is intertwined with the semiconductor sector’s dynamics. Recently, COVID-19 induced cautious supply-side expansion and increased safety stocks, leading to shortages across the semiconductor chain, affecting everything from advanced to mature wafer production capacities. This situation has escalated wafer prices and extended to semiconductor packaging, testing, and circuit design, highlighting the importance of collaboration between suppliers and customers to maintain high-quality wafer production amidst shortages.

The quality of wafers, the foundational material for semiconductors, is determined after numerous manufacturing processes and is influenced by various factors such as equipment, personnel, and timing. Defects often become apparent only after wafers are used by customers, causing significant losses. As semiconductor components become more complex and smaller, the tolerance for metal contamination decreases, leading to potential quality issues [2].

The relationship between white pixel defects in wafers and the crystal lies in the crystalline structure’s quality and the fabrication process. White pixel defects can emerge due to inconsistencies or impurities within the crystal substrate of the wafer, affecting the electrical properties essential for device functionality. High crystal quality, involving purity and structural integrity, is vital to minimize such defects, ensuring the reliability and performance of semiconductor devices [3].

Chien et al. [4] investigated white pixel defects in wafers using convolutional neural networks (CNNs) for the inspection and classification of semiconductor wafer surface defects. Their deep learning approach demonstrates high accuracy in identifying various defect types, offering significant improvements in yield. Ma et al. [5] reviewed the advancements in wafer surface defect detection, emphasizing the role of these technologies in quality control. They categorize detection techniques into image signal processing, machine learning, and deep learning, providing insights into the algorithms’ strengths and limitations. The reviews [6,7,8] also highlight ongoing challenges and future research directions, showcasing the rapid development of neural networks in defect detection, aiming to boost efficiency and accuracy while reducing manual intervention in semiconductor manufacturing.

The main cause of white pixel defects in wafers is contamination from metal impurities and defects from grinding process blades. While it is challenging to reduce metal contamination immediately, addressing blade-induced defects can significantly reduce these issues in CIS products. This study examines how Company F (Taiwan, China), a leading semiconductor wafer manufacturer in Taiwan, tackles white pixel defects, focusing on enhancing response to customer complaints and defect analysis. By employing machine learning techniques, the study aims to improve resource utilization and identify defect causes through reverse engineering. The ultimate goal is to develop a predictive model for white pixel defects, providing process improvement engineers with proactive strategies to minimize these defects.

## 2. Preliminary

### 2.1. White Pixel Defects

CMOS image sensors, extending from office devices to household uses like internet video and reversed-image assistance systems, face issues like noise and low sensitivity due to dark current. By focusing on reducing dark current through failure mode analysis and identifying process-related defects, improvements in the CMOS manufacturing process could make CMOS a viable replacement for CCD. Key findings include high dark current linked to surface damage and defects near specific components, attributed to plasma charging-induced defects, highlighting areas for process improvement.

Defects in CMOS image sensors originate not only from the CMOS process but also from weaknesses in the raw wafer process, leading to intrinsic defects like bulk micro defects due to high oxygen levels. These defects, including dislocation pits, are closely linked to white pixel occurrences. Analyzed customer returns, specifically 300 mm EPI wafers for CIS, highlight metal contamination and the wafer’s internal recovery capability as primary causes of white pixel defects.

**Figure 1:**
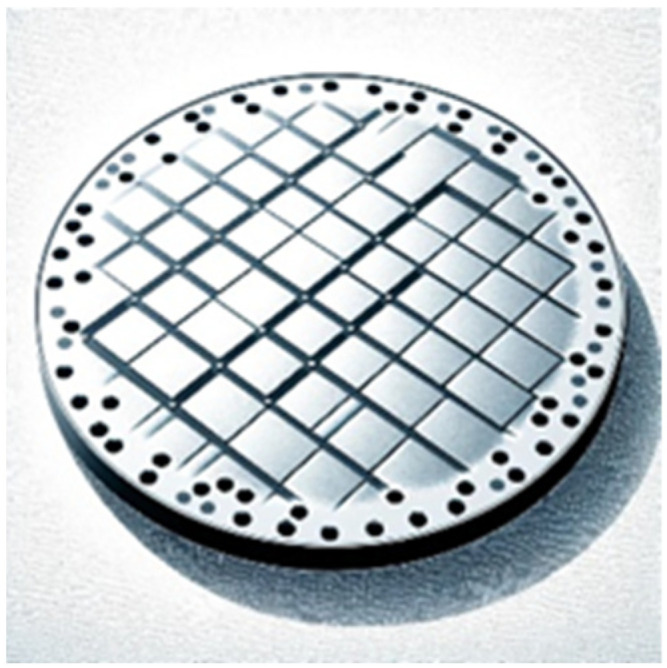
A demonstration of wafer with white pixel defects.

White pixel defects in wafers refer to specific types of imperfections that appear on semiconductor wafers used in the manufacturing of integrated circuits and other electronic devices [9,10]. These defects are visible as white spots or pixels when the wafer is inspected visually or with imaging tools. White pixel defects can result from a variety of sources, including contamination, process deviations, material imperfections, or issues with the equipment used in the semiconductor fabrication process. The presence of these defects can affect the performance, reliability, and yield of semiconductor devices, making it crucial to detect and classify them accurately for quality control and process improvement. Figure 1 is an illustration depicting a semiconductor wafer with white pixel defects. The image shows a close-up view of the wafer, highlighting the white spots or pixels indicating defects scattered irregularly across its surface, providing an insight into how these defects appear in semiconductor manufacturing.

It is not possible to immediately reduce the contamination of metal impurities during the manufacturing process, but improving the defects caused by grinding blades can significantly improve the white pixel defect problem of CIS products. When customers report white pixel issues in CIS products, it is crucial to diagnose the defect’s cause, confirm it with the responsible party, and request an improvement report for customer approval. Insights from discussions with senior engineers from Company F, have identified key factors affecting white pixels in wafer grinding process blades, summarized in Table 1.

### 2.2. Machine Learning Approach

From the perspective of time efficiency, the advantages of machine learning models are as follows:

1. Machine learning models can process large amounts of image data (such as semiconductor wafer scans) faster than manual inspection methods or traditional automated methods that rely on simpler algorithms. Once trained, these models can analyze new imagery almost instantly, which is critical to maintaining production line speeds and meeting high demand.
2. Machine learning models are able to learn from data and improve over time without being explicitly programmed for each specific task. This means they can become more efficient and accurate as more data are processed.
3. The automation provided by machine learning reduces the need for human input during the inspection process, allowing for continuous operations without the interruptions or slowdowns typically seen with manual inspections, and reducing the potential for human error.
4. Machine learning systems can easily scale as production volumes increase and can easily adapt to different types of defects or changes in the manufacturing process. Traditional methods may require significant reconfiguration or redevelopment to handle new types of defects or changes in the manufacturing environment.
5. Machine learning models can be integrated into existing quality control systems to enhance their functionality rather than completely replacing them. This integration can significantly increase speed and efficiency because these models enhance the capabilities of traditional methods by adding layers of intelligence and adaptability.
6. In addition to detecting existing defects, machine learning models can predict potential future failures by identifying subtle patterns that may precede defects. This saves a lot of time and resources.

In summary, machine learning models provide significant time efficiency advantages by automating and accelerating the defect detection process, reducing manual labor, and improving the adaptability and predictive capabilities of semiconductor manufacturing quality control systems.

The choice between basic machine learning models and deep learning models depends on the specific requirements of the task, including the need for interpretability, dataset characteristics, computational resources, and expertise. For white pixel defect prediction, the advantages of basic models in terms of interpretability, efficiency, and performance on structured data make them appealing choices for initial exploration and analysis. Therefore, C5.0 and CART, along with random forest and SVM, are selected in this study to further investigation.

#### 2.2.1. Decision Tree (C5.0)

The decision tree algorithm, known for its high interpretability, constructs predictive machine learning models using straightforward rules. It operates like a Boolean function, processing attributes to output a “yes” or “no” decision. The C5.0 algorithm, recognized for its maturity and widespread use in prediction research, perfectly suits the construction of models for this study’s CIS white pixel issue analysis [11].

#### 2.2.2. CART

The CART algorithm constructs both classification and regression trees. It uses binary splits to divide samples and bases each optimal split on a single variable. For categorical variables, it employs the Gini index or Towing, and for continuous variables, it uses methods like least squares. CART’s simplicity and binary-tree structure make it suitable for both categorical and binary scenarios, with a pruning strategy to refine the model, making it effective for diverse data types and predictive modeling [12].

The CART algorithm is advantageous for its non-reliance on data type assumptions, its capability to manage complex data, and its flexibility in not pre-defining rules, allowing for easy information retrieval. Its key strength lies in developing fully grown trees optimized via pruning, making it ideal for predictive modeling across various domains. Given its maturity and widespread application in predictive research, CART is selected for modeling the CIS white pixel issue in this study, demonstrating its suitability for addressing complex analytical challenges.

#### 2.2.3. Random Forest

The random forest (RF) algorithm, originating from Bell Labs in 1995, merges multiple decision trees to form a collective decision based on the majority. It is celebrated for its accuracy, ability to handle numerous input variables, and versatility across various data sets. RF estimates missing data, ranks variable importance, and detects variable interactions, useful for outlier detection and data visualization. While efficient, RF’s performance might suffer from computational demands or noise in data, potentially leading to overfitting [13].

#### 2.2.4. Support Vector Machine

The support vector machine (SVM) algorithm is a versatile supervised learning method that excels in classifying data into distinct categories by finding the optimal separating hyperplane. It is particularly effective for high-dimensional spaces and situations where the number of dimensions exceeds the number of samples. However, SVM can be computationally intensive for large datasets and may struggle with noisy data. Additionally, SVM does not inherently provide probability estimates, requiring additional steps like five-fold cross-validation to obtain these.

## 3. Methodology

This study addresses semiconductor quality issues through the application of various tools and techniques. In particular, machine learning technology is employed to develop a prediction model for identifying white pixel defects. Decision tree algorithms such as C5.0 and CART, along with random forest and SVM, are utilized in the R programming language to construct the prediction model. This section provides a detailed explanation of the research methodology.

### 3.1. Framework

**Figure 2:**
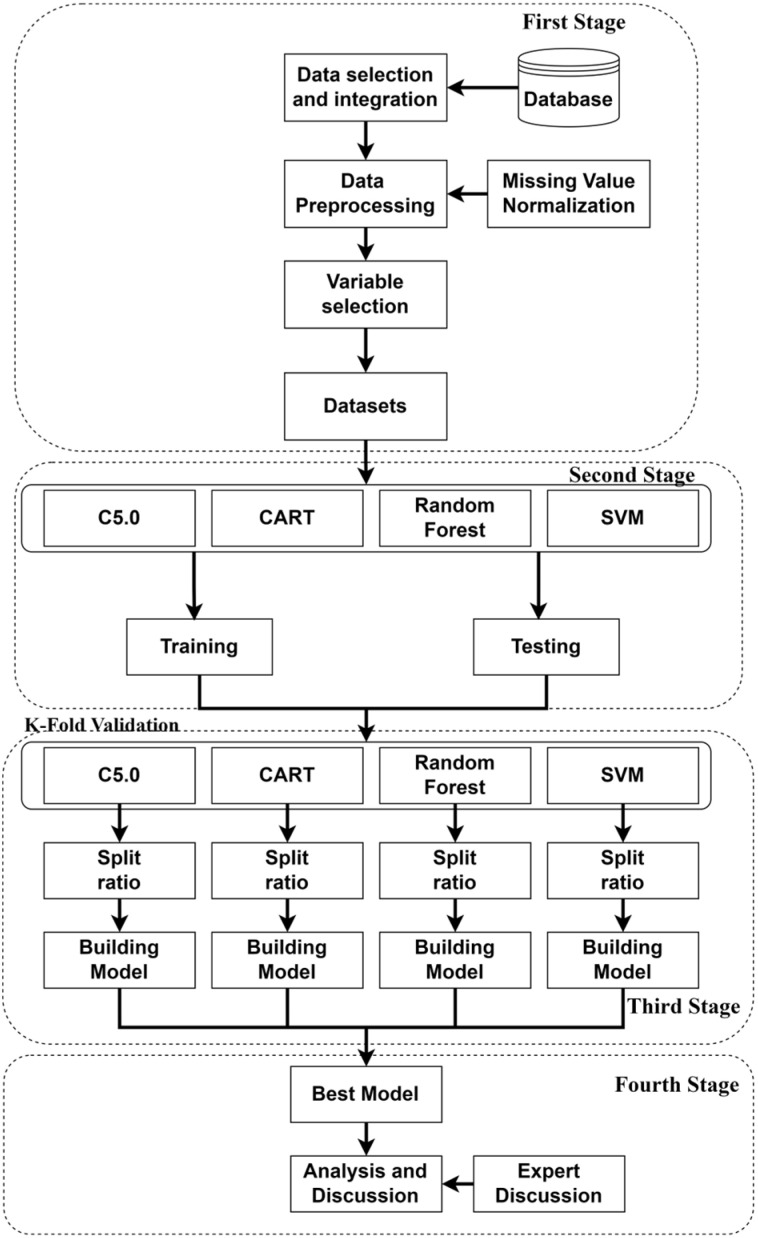
Framework of the study.

The study’s framework, as illustrated in Figure 2, involves four key stages, each contributing to the construction and evaluation of the predictive model.

1. First Stage—Data Selection and Integration: This stage involves outlining the data sources utilized in the study, along with a description of how the collected data are processed and formatted to meet the study’s requirements. It includes defining the research dependent and independent variables. Data pre-processing: here, the study addresses noise reduction, elimination of incomplete data, and normalization techniques to enhance the accuracy of the predictive model. Research variable screening: the methodology for variable selection is explained, including the use of a multinomial logit regression algorithm in R to identify significant factors influencing white pixel defects, resulting in the construction of the input model with 45 relevant variables.
2. Second Stage: At this stage, the basic model of machine learning is selected. The choice between basic machine learning models and deep learning models depends on the specific requirements of the task, including the need for interpretability, dataset characteristics, computational resources, and expertise. For white pixel defect prediction, the advantages of basic models in terms of interpretability, efficiency, and performance on structured data make them appealing choices for initial exploration and analysis. Therefore, C5.0 and CART, along with random forest and SVM, are selected to further the investigation.
3. Third Stage: In this stage, the analyzed data are employed for model construction and prediction procedures. The K-fold cross-validation method is employed to identify the most accurate prediction model.
4. Fourth Stage: To compare and evaluate the R language-constructed models, the study fixes the seed setting value at 123 in R for consistency across calculations. The decision tree (C5.0), CART, RF, and SVM models are constructed and compared. The best-performing prediction model is revealed. Furthermore, significant factors obtained from models are extracted and analyzed for their impact on white pixel defects.

### 3.2. Case Study

**Figure 3:**
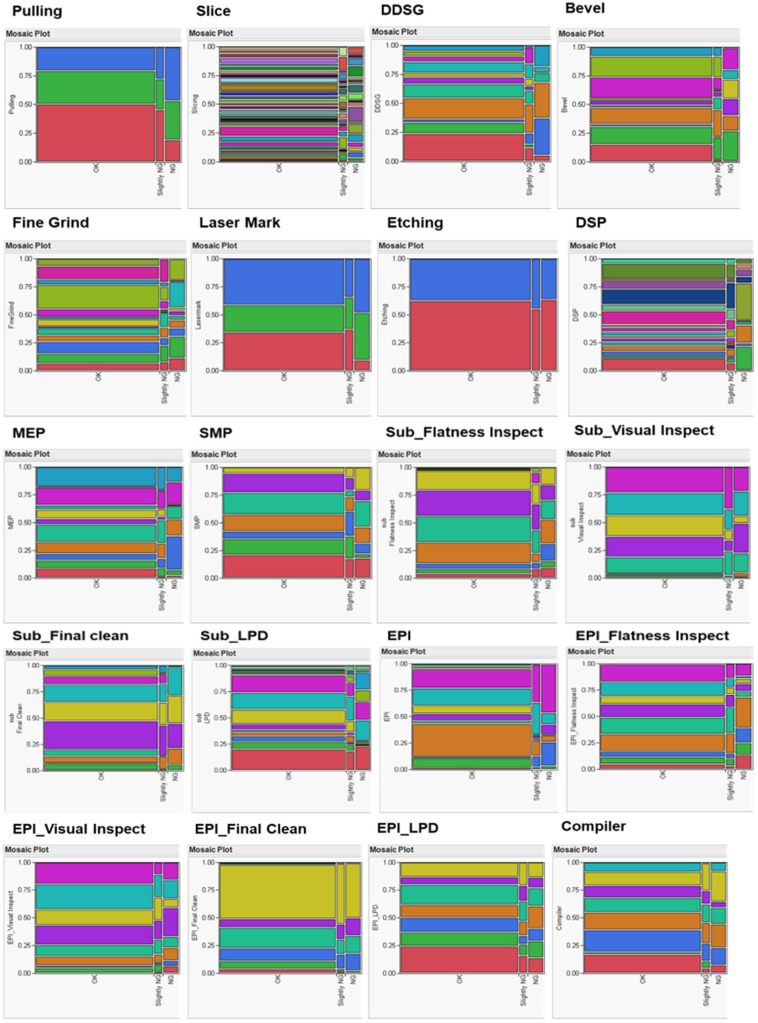
Confirmation of equipment commonality (source: Company F).

This case pertains to Company F, a silicon wafer manufacturing firm located in central and southern Taiwan. In 2019, the company faced a significant issue when white pixel defects were detected on client-side wafers, leading to a decline in the final product yield. Typically, white pixel defects can stem from various factors such as metal contamination on the wafer or internal defects within the wafer layers. Sources of wafer contamination and defects may include unclean processing equipment, contaminated materials, or inadequate clean room conditions [14,15]. Additionally, environmental impurities or abnormal processing parameters of the wafer material itself can contribute to such issues. These challenges are ubiquitous across wafer manufacturing plants, affecting both upstream and downstream clients alike. In this particular case, Company F received reports from customers indicating abnormally high rates of white pixel defects across different products, with a defect rate soaring as high as 80%—a stark contrast to the typical rate of around 10%, as depicted in Table 2 below. Due to the widespread presence of white pixels covering the entire surface of the wafer, they were compelled to halt shipments to prevent further losses. This decision placed immense pressure on Company F’s shipping operations. Consequently, addressing the white pixel issue promptly and effectively became paramount to mitigate any prolonged disruption. Upon scrutinizing Company F’s processing equipment records, it was observed that wafers exhibiting white pixels did not share the same processing number, as illustrated in Figure 3.

**Figure 4:**
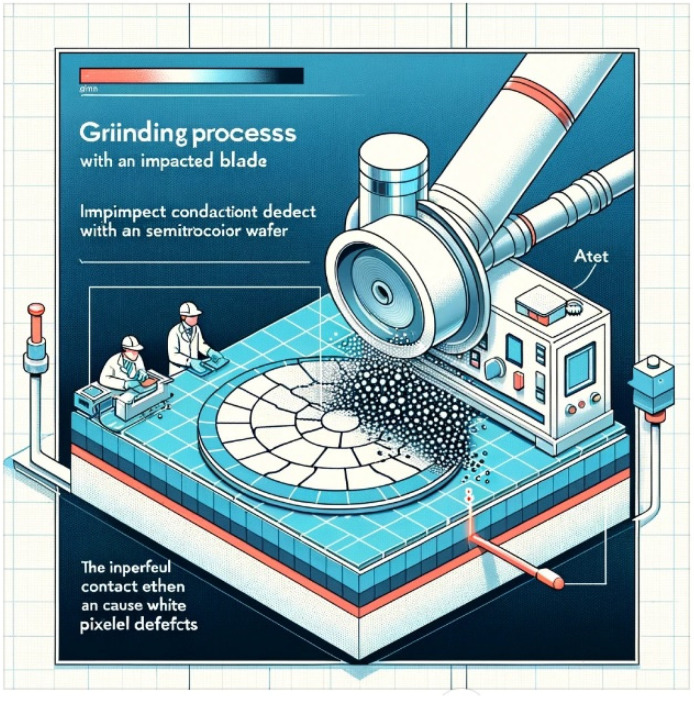
A simulated impacted blade leads to white pixel defects in a wafer.

**Figure 5:**
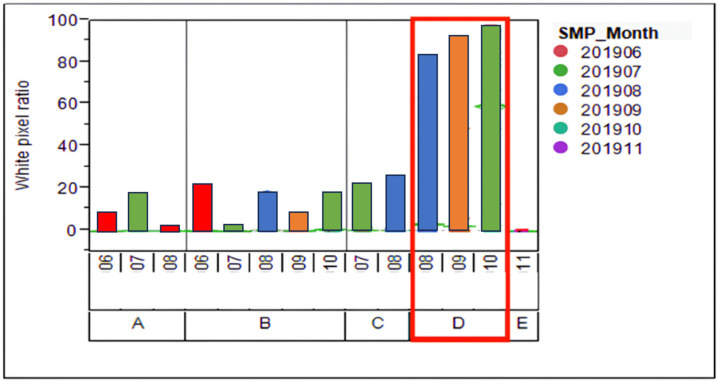
Survey on pad material numbers used in leaf grinding machines (Redbox means abnormal increase in wafer white pixels).

Due to the company need to immediately resolve the white pixel defect problem, this study focuses solely on the impacted blade grinding process. Upon further investigation, Company F confirmed that all materials and products used in the grinding equipment contributed to surface defects on the wafers. Figure 4 is a simulated diagram illustrating how the grinding process with an impacted blade can lead to the formation of white pixel defects in a semiconductor wafer. The visualization shows the side view of the grinding process, highlighting the relationship between the condition of the blade and the occurrence of defects on the wafer. It was noted that, starting from August 2019, following processing on Pad D, there was a notably higher incidence of white pixel abnormalities observed on the client side compared to other pads. This led to the realization that the abnormal white pixels reported by customers were strongly linked to Pad D in Company F’s grinding machine, as depicted in Figure 5. However, there remains a possibility that other pads may also be affected, albeit undetected thus far. This anomaly has instilled distrust among customers towards Company F’s products, prompting them to cease shipments. Without appropriate intervention, this situation could lead to unforeseeable losses for Company F, including potential customer compensation. Hence, addressing the white pixel problem is imperative and must be prioritized as a top concern.

**Figure 6:**
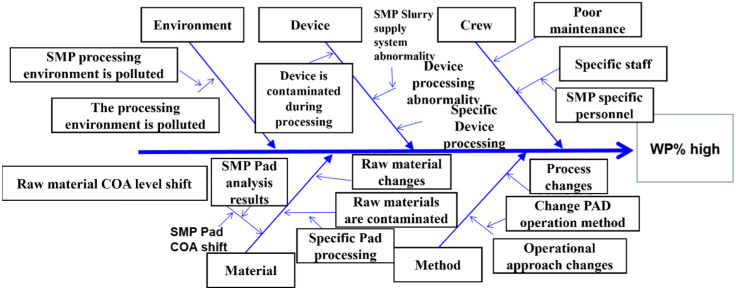
Factor analysis of white pixels in wafer manufacturing plants.

Currently, Company F employs cause analysis and identifying the root cause (D4: Identify the Root Cause) within Ford’s 8D approach to pinpoint potential influencing factors. After expert inferences and experimentation, and subsequent analysis by quality and technical engineers, consistent results have been obtained. It is concluded that the manufacturing parameters of the pad utilized in the grinding equipment should be adjusted, as they are believed to directly impact the product quality during processing. The analysis results are outlined in Figure 6 below.

**Figure 7:**
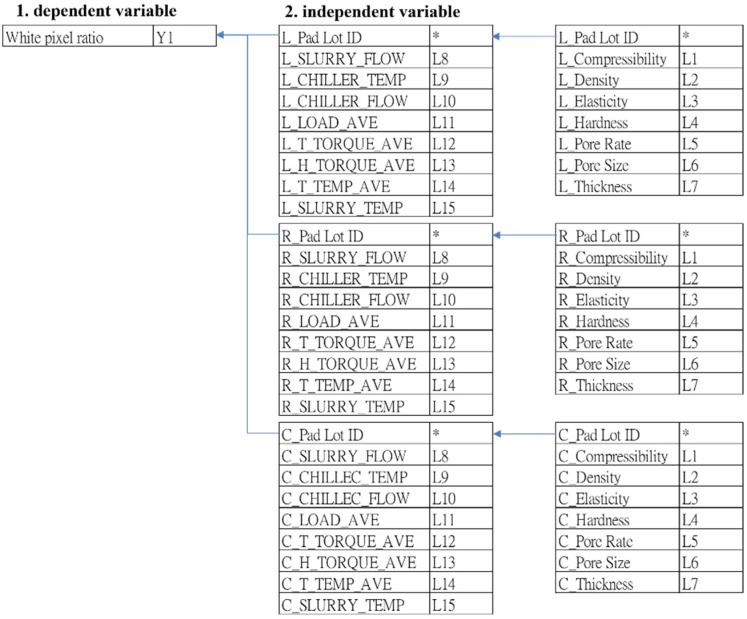
The relationship between dependent variables and independent variables (*: no value).

The data collection period for this study spans from April 2019 to the end of November 2019, totaling 4980 pieces. Subsequently, the following steps were undertaken to organize the dataset:

- Step 1: Gather 4980 pieces of Company F’s process data and customer complaint pixel data, focusing on significant periods for analysis.
- Step 2: Define model variables. Following discussions with senior engineers specializing in wafer manufacturing technology, the research scope was confined to the wafer surface grinding process. The aim was to identify input variables potentially affecting white pixel defects in CIS products.
- Step 3: Screen processing variables of the grinding process. The grinding mechanism comprises three programs: R, C, and L. Within the wafer mirror grinding process, the R program aims to eliminate grinding stress and remove oxide film, while the C and L programs aim to enhance surface roughness and particle levels. The RCL program revealed 45 influencing variables, each described in Table 3, with their interrelations and correlations depicted in Figure 7.
- Step 4: Data pre-processing. After confirming the research range of the collected case data and deleting missing data and duplicate data.

#### Data Collection and Pre-Processing

- Data Attributes: The data collection for this study revolves around information concerning the pad of Company F’s grinding equipment. The primary focus is on predicting its degree of influence on white pixels, serving as the dependent variable. This determination is based on the processing conditions of the grinding equipment and the material certificate of analysis (COA) information. The white pixel result information provided by Company F’s customers is categorized into groups, as outlined in Table 4. It is classified into two levels: ‘OK’ indicating good products and ‘NG’ indicating seriously defective ones.
- Variable Selection: The select variables in this study involved consultations with senior engineers from Company F. All polishing engineering variables potentially impacting the white pixel problem were chosen based on the processing conditions of the individual wafer manufacturing plant. The suggested items are detailed in Table 5, comprising a total of 45 variables utilized as initial input variables.

This study categorizes variables into two types: OK and NG. Initially, significant factor testing is conducted on the data comprising 45 variables. The multinomial logit regression model is utilized as an exploratory tool for factor testing. Logit regression testing primarily assesses the statistical hypothesis regarding the presence of a significant correlation between the variable and the dependent variable. The Wald test is employed to determine the significance of independent variables. With 1 degree of freedom, the critical value of X^2^ for α = 0.05 is 3.841. When a particular independent variable demonstrates significance, the Wald statistic value exceeds 3.841. This criterion is utilized in this study for selecting input variables.

### 3.3. Data Preprocessing

Before embarking on data exploration, it is essential to preprocess the large datasets to make them more amenable for analysis and model building. This involves preparing the data through cleaning, quantifying, and normalizing to fit the required format specifications. Data preprocessing can address the following issues:

1. Handling Incomplete or Noisy Data: It is common to encounter missing fields or incomplete data within a dataset. To address this, one approach is to outright delete the entries or to replace them with the average value of the dataset. Alternatively, setting missing values to a null state allows for their exclusion from analysis.
2. Data Normalization: To ensure that the dataset aligns with the requirements of the predictive algorithm, it must undergo normalization. This process adjusts input and output variables to fit within a specific range, often called scaling. After model training, the output values produced during inference need to be rescaled back to their original range. This study employs interval mapping for scaling, adjusting the data variables’ minimum and maximum values to align with the desired range. As a result of normalization, all input values are scaled to fall between 0 and 1.

### 3.4. K-Fold Cross-Validation

The K-fold cross-validation method is employed in this study for sample partitioning when constructing a prediction model. Typically, collected samples are divided into a training set and a test data set (Test Set), with each sample individually separated [16]. Initially, the training data set is utilized to train a model, followed by testing its performance using the test data set to assess model consistency [17]. However, the size of the data sample can influence the proportion of training and test data, consequently impacting the performance of the built model. To address this, the study utilizes K-fold cross-validation.

### 3.5. Evaluation Metrics

Through the following evaluation metrics, the effectiveness obtained after classification can be evaluated, and the classification results can be divided into true positive (*TF*), that is, correct acceptance, true negative (*TN*) or correct rejection, false positive (*FP*) or wrong acceptance, and false negative (*FN*) or wrong rejection. Accuracy, sensitivity, specificity, and other relevant metrics can be calculated from these four metrics [18,19].

(1) Accuracy

An accuracy assessment is defined as the ratio of correctly predicted samples to the total number of predicted samples. *TP* is true positive, *TN* is true negative, and total represents the total number of predictions.

(1) $$Accuracy=\left(\frac{\left(TP+TN\right)}{Total}\right)$$

(2) Precision

Precision calculates the correct classification number that is penalized for an incorrect classification number.

(2) $$Precision=\frac{TP}{TP+FP}$$

(3) Specificity

Specificity is the proportion of people without an original negative test, commonly referred to as the proportion of “true negative”.

(3) $$Specificity=\frac{TN}{FP+TN}$$

(4) F-Score

F-Score is the harmonic average of measurement accuracy and recall rate.

(4) $$F\text{-}Score=2\times \frac{Precision\times Recall}{Precision+Recall}$$

Choosing overall accuracy as the performance evaluation metric for white pixel defect prediction in semiconductor wafers might not always capture the full picture. However, overall accuracy might be initially appealing or chosen as a primary metric in simplicity and understandability and initial benchmarking for director of Company F.

## 4. Experimental Results and Analysis

**Figure 8:**
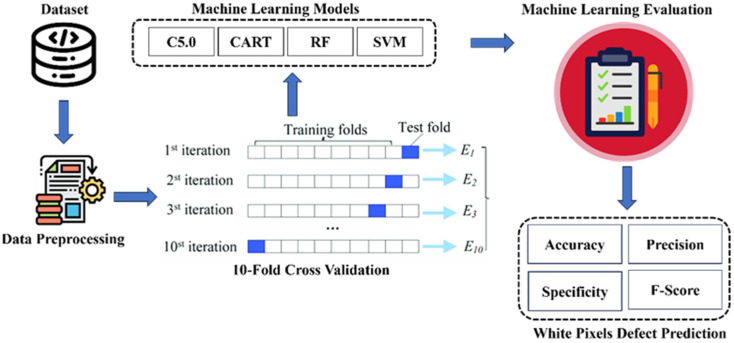
Block diagram of the experimental process.

This study employs the Python scikit-learn library across four steps: preprocessing data, training, and testing with 10-fold cross validation in four machine learning models as shown in Figure 8. Data preprocessing addresses missing values and imbalance. Models such as decision tree (C5.0), CART, RF, and SVM were trained, tested, and evaluated for metrics such as accuracy, precision, specificity, and F-score in white pixel defect prediction.

Below is the detailed experimental environment for this study:

1. Hardware: Powerful CPU (Intel i9), high-performance GPU (NVIDIA RTX 3080), ample RAM (32 GB). Fast solid-state drive (NVMe SSD).
2. Software: GUI and compatibility for the operating system Windows. Programming language: scikit-learn, R4.3. Scikit-Learn 1.4 for general machine learning. Git is used for source code management. GitHub online code repository and collaboration.
3. This comprehensive environment supports a wide range of machine learning activities from data preprocessing and model training to deployment and monitoring, ensuring experiments can run efficiently and effectively.
4. It is a common practice to use default parameter values when comparing multiple machine learning models, especially in the initial stages of model evaluation. There are usually several reasons for this approach:
5. Default parameters provide baseline performance for each model. This allows you to immediately see the performance of each algorithm without making any adjustments. This is a quick way to measure how well a model suits a particular type of data or problem.
6. Using default settings, all models start on a level playing field. This fairness is crucial when your goal is to compare different types of models to understand which model inherently fits the material better and is not subject to extensive parameter optimization.
7. Use preset parameters to make your experiments more reproducible. Other researchers can easily validate your results by using the same model with default settings, ensuring that performance metrics are attributed to the model itself and not to specific adjustments made to it.

The initial performance of default parameters can guide more targeted hyperpa-rameter tuning in the future. It might be worth tweaking if the model shows good promise under the defaults. Conversely, if a model performs poorly in its default settings, it may be lowered in priority or require significant changes to its settings. It is important to realize that while default parameters are useful for initial comparisons, they are rarely optimal for any given task.

### 4.1. Sample

**Figure 9:**
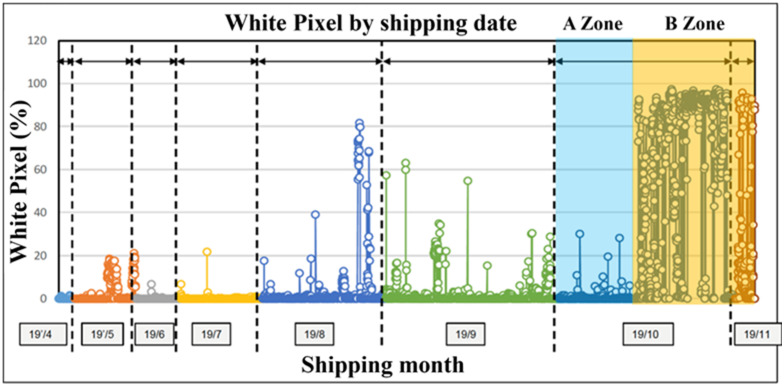
White pixel occurring period.

The total quantity of data collected for this research is 4980. To explore the predictive ability when white pixel defects occur, the data are constrained to the period from October 2019 to November 2019—both before and after the occurrence of white pixels. The primary distinction is made between the A Zone (before a significant number of white pixel defects occurred, totaling 1152 cases) and the B Zone (after a significant number of white pixel defects occurred, totaling 910 cases) as shown in Figure 9.

### 4.2. Variable Selection and Results

This study delineates its scope into two distinct phases: the period preceding the abnormal increase in white pixels (the A Zone) and the phase during which an abnormality in white pixels is observed (the B Zone).

1. Screening Variables in the A Zone (Count: 1152): The investigation utilized 45 variables, recommended for assessing the processing conditions within a semiconductor wafer manufacturing facility. These variables served as input factors for conducting a screening process. Utilizing the logistic regression algorithm [20], implemented in R programming language, the study aimed to identify variables significantly influencing the occurrence of white pixel defects. The analysis, focusing on the period before the notable rise in white pixel defects (A Zone), revealed that six variables significantly impact the incidence of these defects. These variables are distributed across three axes: two significant variables on the L-axis, two on the R-axis, and two on the C-axis, as detailed in Table 6.
2. Variable screening in the B Zone: In the B Zone (Count: 910, where a large number of white pixel defects occur), 27 significant items of variable (L-axis: 7 items, R-axis: 11 items, C-axis: 9 items) were found, as shown in Table 7.

Since the 27 important variables in the B Zone include all important characteristic variables in the A Zone, this study uses all important variables in the B Zone as input variables to proceed with machine learning. Each machine learning model written in the R language is trained and predicted using data from these areas, and subsequently, the area with higher accuracy in predicting white pixel defects is observed, as depicted in Figure 8.

**Figure 10:**
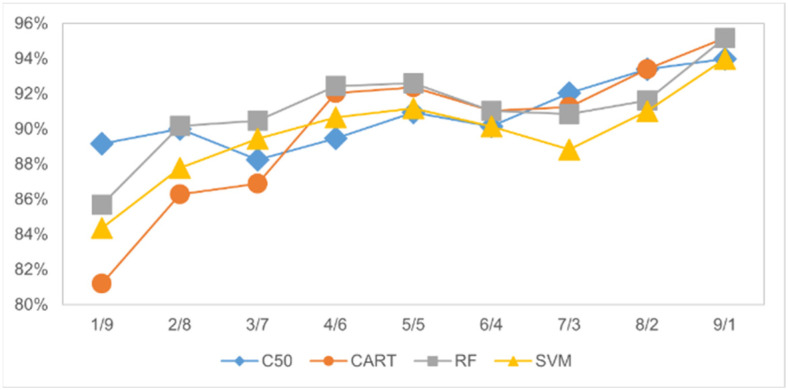
Average accuracy of distribution of training and test sample proportions.

Initially, the data sample set is divided into training and test sample sets after determining the number of samples for each overlap. Using the R language, the study seeks to identify the most suitable proportion between the training and test sample sets. The total data from the A Zone and B Zone (totaling 2062 records) serves as the basis. Each model is then verified using sequential allocation ratios between the two areas, ranging from 1:9 to 9:1. The results, evaluated in Table 8 and shown in Figure 10 below, assume that the best accuracy can be obtained in individual models when the training-to-test ratio is 9:1 by a 10-fold cross validation average.

It can be seen from Figure 10 that as the proportion of the test set becomes smaller, the average accuracy rate also decreases. And, the CART and RF models have the highest accuracy (95.18%) among the four machine learning models in the default parameter setting. The evaluation metrics of each model are also shown in Table 9 for comparison.

### 4.3. Comparison of Prediction Models for the A and B Zones

This study not only examines the entire sample interval but also seeks to examine if the accuracy of the predictive models varies between two distinct A and B Zones.

The study evaluated four predictive models: the decision tree C5.0, CART, RF, and SVM at the A Zone in the default parameter setting. The division between training and testing data within the K-fold cross-validation method adhered to a ratio based on the training sample set, consisting of nine groups, with the testing sample set comprising one group. As a result, in Table 10, the RF model outperformed the others in terms of average prediction accuracy, achieving a training accuracy rate of 94.206% and a testing accuracy rate of 94.940%. The models are ranked by their accuracy as follows: RF, CART, and support vector machine (SVM), as illustrated in Table 10.

Then, the data from the B Zone are introduced into the four machine learning models in the default parameter setting. The prediction results are shown in Table 11. Among the four models, the prediction accuracy ranking using a 10-fold cross-validation average are ranked by their accuracy as follows: RF, decision tree (C5.0), CART, and SVM.

**Figure 11:**
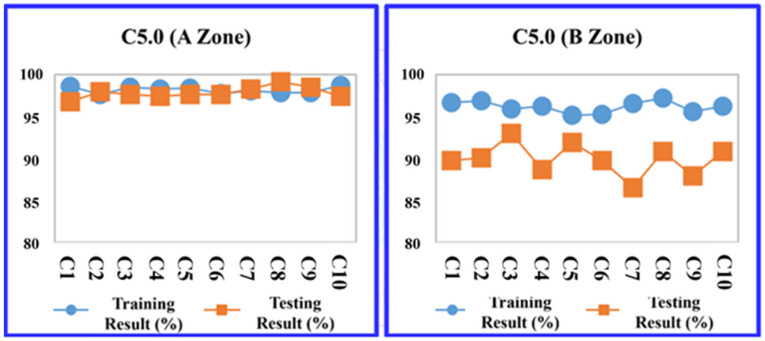
Decision tree (C5.0) average accuracy transition chart (A and B Zones).

**Figure 12:**
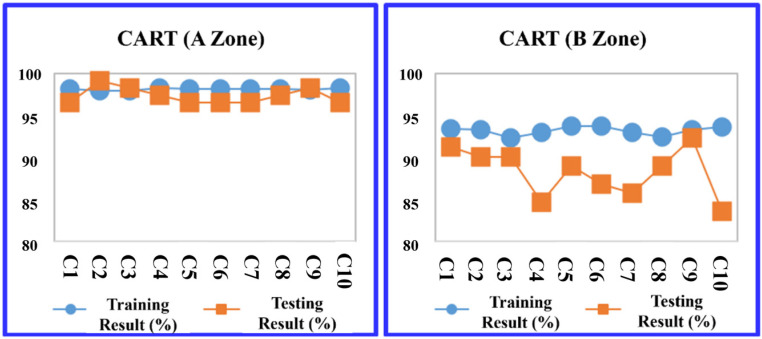
CART average accuracy transition chart (A and B Zones).

**Figure 13:**
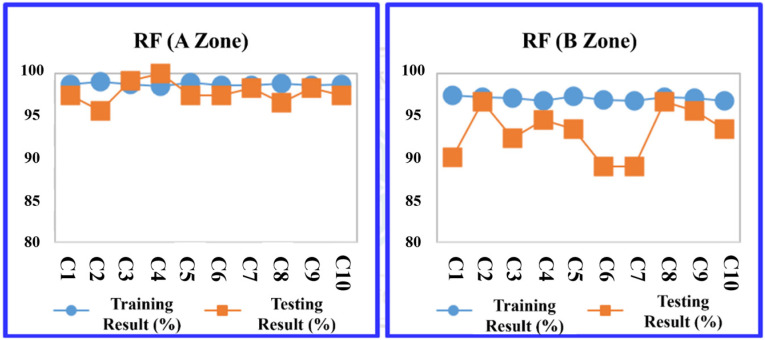
RF average accuracy transition chart (A and B Zones).

**Figure 14:**
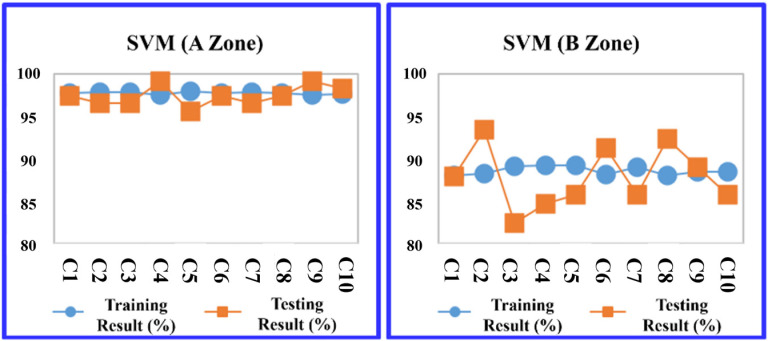
SVM average accuracy transition chart (A and B Zones).

More detailed training and testing results of the four models are shown in Figure 11, Figure 12, Figure 13 and Figure 14.

### 4.4. Prediction Rule Extraction and Factors Discussion

In Section 4, the study revealed that CART and RF are better models for the prediction of white pixel defects. Consequently, the focus of this evaluation and comparison will be on the RF and CART models. Analysis will proceed with CART’s more recent algorithm for in-depth discussion.

**Figure 15:**
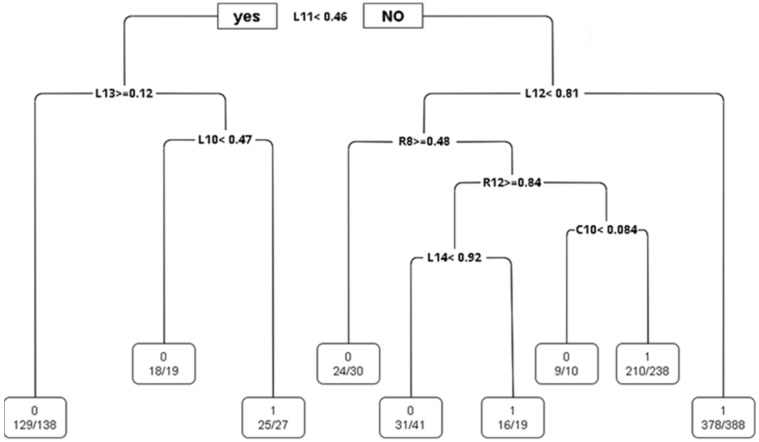
Visualization of the CART decision tree (before pruning).

**Figure 16:**
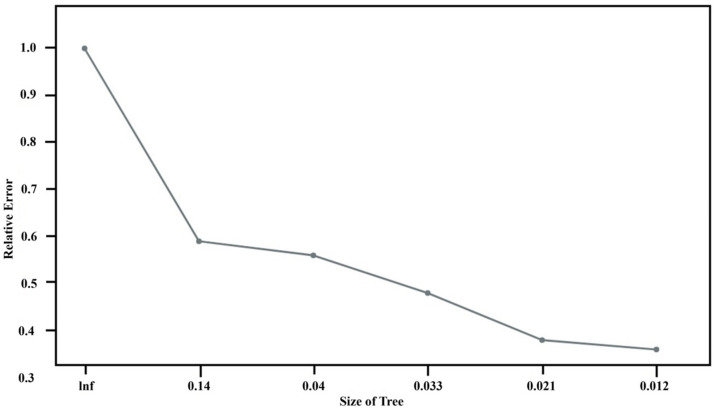
Changes in the error rate of the fitted model.

1. CART: The study employs the CART algorithm for the development of a decision tree model, as illustrated in Figure 15. An analysis of the complexity parameters and error rates for the unpruned CART decision tree model post-training reveals insights into the model’s error dynamics. As demonstrated in Figure 16, the model achieves its minimum cross-validation error of 0.013780 when the tree is configured with nine leaf nodes (indicating eight splits).

**Figure 17:**
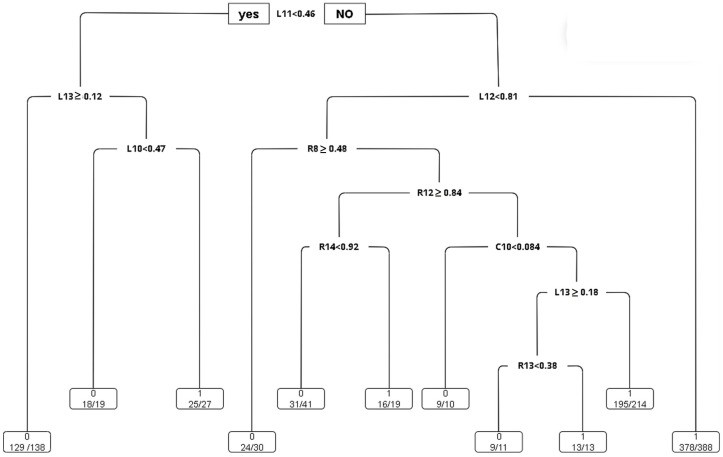
Visualization of the CART decision tree (after pruning).

In this study, the R language prune is used to prune the CART decision tree. The cp value is set to 0.021. The results can be obtained from the visual CART decision tree (as shown in Figure 17). It is useful for distinguishing whether there are white pixel defects. The important indicators are C10, L10, L11, L12, L13, R12, R14, and R8, respectively.

Figure 17 presents the CART decision tree, revealing that L_Load_AVE (L11) emerges as a critical determinant, succeeded in importance by L_T_TORQUE_AVE (L12) and L_H_TORQUE (L13). The rules extracted from the CART decision tree are concisely summarized in Table 12. For example, when L11(L_LOAD_AVE) < 0.46 and L12(L_T_TORQUE_AVE) ≥ 0.81, the probability of white pixels is 97%; when L11(L_LOAD_AVE) < 0.46 and L13(L_H_TORQUE_AVE) ≥ 0.12, the probability of no white pixel defect is 94%, and so on.

2. RF: The mean decrease Gini coefficient, often referred to in the context of random forest and other tree-based models, measures the importance of a feature (variable) in a predictive model. A higher mean decrease Gini indicates that a feature more effectively splits the dataset into groups with similar outcomes, thus being more important for the prediction. The trained random forest model can measure the importance of variables through the mean decrease Gini coefficient. The data show that variable L11 is the most important feature, followed by R11, L12, C10, R8, L13, and R14.

In addition, SHAP is a game theory approach used to explain the output of any machine learning model. It assigns each feature an importance value for a particular prediction, which helps in interpreting the model’s decision-making process. The key idea behind SHAP is to use Shapley values from cooperative game theory to fairly distribute the prediction among the features.

Shapley values provide a weighted average of the marginal contributions of each feature across all possible feature combinations. This method ensures a consistent and fair attribution of importance to each feature, accounting for interactions among features within the model. By applying SHAP values, one can gain insights into how each feature contributes to the prediction, regardless of the model complexity.

**Figure 18:**
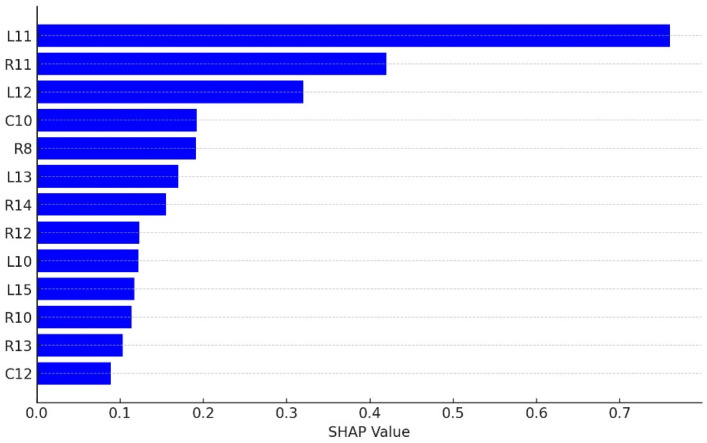
SHAP values of features.

Figure 18 is a SHAP diagram illustrating the Shapley values for top 12 features. The features are sorted by their absolute Shapley values, highlighting their impact on the model’s prediction. This visualization helps in understanding the relative importance of each feature in the model’s decision-making process.

We summarize the ranking of important factors from the CART and RF models and shown in Table 13, In the feature verification of CART and RF, except for the R11 item, the other common feature items are regarded as important factors by RF and CART, and the L-axis torsion (L11) is the most important factor that mainly affects the occurrence of white pixel defects.

The L-table grinding load (L11) is considered a major reason for white pixel defects in semiconductor wafers due to several factors associated with the grinding process used in wafer manufacturing in this study. There may be several possibilities as to why high or uneven L-table grinding loads can lead to white pixel defects in wafers:

1. Mechanical Stress: Grinding involves applying mechanical stress to thin the wafer to the desired thickness. An excessive load during this process can induce micro-cracks and subsurface damage, which may not be visible immediately but can manifest as white pixel defects later in the manufacturing process or during the operation of the final semiconductor device.
2. Heat Generation: Grinding generates heat due to friction between the wafer and the grinding wheel. High grinding loads increase this heat generation, potentially causing local overheating. This can lead to slip dislocations, changes in material properties, or other forms of damage at a microscopic level, contributing to defect formation.
3. Surface Quality Degradation: The quality of the wafer surface post-grinding is crucial for subsequent manufacturing steps, such as lithography. High grinding loads can degrade surface quality by introducing roughness, pits, and scratches. These surface irregularities can interfere with subsequent processes, leading to defects, including white pixels, in the finished product.
4. Impurities and Contamination: The grinding process, especially under high loads, can lead to the embedding of abrasive particles or the generation of debris that becomes embedded in the wafer surface. These impurities can act as nucleation sites for defects.
5. Non-uniform Material Removal: Ideally, grinding should remove material uniformly across the wafer. However, excessive or unevenly distributed grinding loads can lead to non-uniform thickness, warping, or localized thinning, which may result in stress concentrations. These stress concentrations can manifest as white pixel defects during further processing or in the final product.

Mitigating these issues requires careful control of the grinding process, including optimizing grinding parameters like load, speed, and coolant flow, to ensure the mechanical stresses and heat generated do not exceed the material’s tolerance thresholds. Additionally, regular maintenance of grinding equipment to ensure uniform contact and load distribution across the wafer surface is essential in minimizing the risk of white pixel defects.

In addition, based on the findings of Das [21], the significance of variables in the CART model is determined by summing up the scores of split improvements for each variable across all tree splits. This method evaluates the impact of variables within the model. On the other hand, the random forest methodology constructs multiple CART trees utilizing bootstrap samples of the data, subsequently aggregating their predictions. This aggregation is typically performed by calculating the average prediction across all CART models within the forest. Random forests are known to offer enhanced predictive capabilities and accuracy compared to a single CART model, primarily due to their reduced variance. These insights align with the results observed in the current study.

## 5. Conclusions

This study focuses on improving CMOS image sensor (CIS) products used in mobiles and cameras by addressing white pixel defects, mainly caused by metal impurities during wafer production. A case study from a Taiwanese silicon wafer manufacturer identifies key factors influencing defect ratios using data analysis. The Cart and RF models proved most accurate in predicting defects with default parameter setting, highlighting some processing load and torque factors as significant factors. These insights offer guidance for preventive measures to reduce defects and customer complaints.

### 5.1. Findings

This study applied various analytical methods, including random forest (RF), decision tree (C5.0), classification and regression tree (CART), and support vector machine (SVM), to investigate risk factors for white pixel defects in semiconductor wafer data. By harnessing machine learning techniques, the goal was to distill meaningful characteristics for a predictive model to forecast white pixel defects on wafers.

The investigation mainly developed an optimal predictive model using RF and CART methodologies. An initial analysis of 45 factors provided by Company F via multinomial logistic regression revealed 27 significant factors. Subsequently, these factors were integrated into both the RF and CART models for additional analysis, pinpointing around 10 significant factors with consistent results across the models. Among these, load and torque related to the L-axis during manufacturing emerged as critical for the onset of white pixel defects.

Discussions with senior technical engineers validated the significance of these factors and the practicality of the study’s findings, underscoring the real-world relevance of the identified critical factors in diagnosing the root causes of white pixel defects. The research offers actionable insights, providing decision-makers with interpretable guidelines, notably:

1. Loadbearing (L11, R11) and torque (C10, L12, L13, R12) during the wafer manufacturing process are identified as key elements. The study indicates a direct relationship between the grinding machinery’s applied load and torque and the probability of causing wafer surface damage, which subsequently increases the risk of white pixel defects. This damage aids in the adherence of external contaminants on the wafer surface, which subsequent cleaning processes may not fully remove. These contaminants, if left on the wafer surface, can infiltrate deeper layers during thermal processing, potentially culminating in white pixel defects in the final product.
2. The study delineates two essential rules for predicting white pixel defects:
  - A higher likelihood of white pixel defects occurs when the SMP L-axis load value meets or exceeds 0.46 and the L-axis Table torque value meets or exceeds 0.81, with the CART model showcasing a 97% accuracy rate in this prediction.
  - Conversely, the probability of white pixel defects markedly decreases when the SMP L-axis load value is less than 0.46, the L-axis chill flow is below 0.47, and the L-axis head table torque value is under 0.12.
3. The analysis further reveals that the most critical factors predominantly pertain to the L-axis, consistent with Company F’s observations. The significant presence of white pixel defects was linked to the L-axis of the SMP grinding equipment, particularly associated with using a specific batch of pads and certain grinding parameters. These factors, related to both the equipment and materials used, were identified as the primary contributors to the notable occurrence of white pixel defects.

### 5.2. Suggestion and Future Study

In the context of CIS products, the occurrence of white pixel defects is influenced by a multitude of factors. This study’s sample focused on analyzing the processing conditions of Company F’s SMP grinding machine and the physical properties of the pad’s raw materials. Although processing conditions were found to have a significant impact, the accuracy of the data provided by the suppliers warrants closer examination. Suppliers often report values close to the detection limit, leading to a uniformity in the raw material data that may cause inaccuracies in model predictions and diminish their predictive capabilities. There is also a study [22] that focuses on the major achievements of CMOS image sensors in surveillance systems in different fields, which is also worth referencing.

In the semiconductor industry, swiftly identifying the relationships between various factors or between factors and anomalies is crucial for improving process efficiency or product yield. Beyond the methodologies applied in this research, employing correlation law analysis or deep neural networks (DNNs), which represent the forefront of AI technology, could offer valuable insights. By initially determining the correlation degrees between factors and then dedicating resources to further analysis and research, it is possible to achieve significant advancements in research directions and outcomes.

## Figures and Tables

**Table 1:** Characteristic comparison of white pixels and wafer fabrication process factors.

Phenomenon	Effect Feature	Quality Features	Engineering	Effect Factor
Abnormal white pixels	Damage	Minor scratch	Grind	Polishing pressure changes
				Pad Lot
				Slurry Lot
	Metal	Bulk layer metal	Crystallization	Contamination within the device
				Material anomaly
			Double-edging polishing	Pad Lot
				Slurry Lot
				Carrier Lot
			Single-wafer polishing	Pad Lot
				Slurry Lot
			Wash after epitaxial	Cleaning liquid cleanliness
				Cleaning liquid concentration
				Environment
			Epitaxy	Equipment abnormality
				Gas cleanliness
				Contamination in the furnace
	Gettering ability of heavy metal pollution	BMD	Crystallization	Crystal pulling condition
				Bron concentration

**Table 2:** White pixel abnormality occurrence ratio.

Product	White Pixel (%)	Output	WIP
EU17	79.5	13L/325pcs	3L/75pcs
EU22		7/175pcs	1L/25pcs
EU11		4L/100pcs	0
EU16	36.5	13L/25pcs	8L/200pcs
EU19		12/300pcs	0
EU25	13.5	13L/325pcs	5L/125pcs
EU30	24.6	21L/525pcs	3L/75pcs
EU33	68.6	71L/1775pcs	8L/200pcs
EU34		19/475pcs	0
EU37	0.3	2L/50pcs	10L/250pcs
EU39		1L/25pcs	4L/100pcs
EU42		1L/25pcs	0
EU18		0	4L/100pcs
		177L/4425pcs	46L/1150pcs

**Table 3:** Description of input variables.

ID	LM_ID	Define Content	Value Type	Impact
Y1	White pixel ratio	Customer response white pixel value	quantitative, qualitative	Affects end-user yield rate
L1	L_Compressibility	Compression ratio of the grinding machine’s L-axis grinding cloth	quantitative	Whether the shrinkage ratio of the grinding process affects the white pixels
L2	L_Density	The space of the grinding cloth on the L-axis of the grinding machine	quantitative	Does the density of the grinding process affect the white pixels?
L3	L_Elasticity	Elastic modulus of the grinding cloth for the L-axis of the grinding machine	quantitative	Does the elastic modulus during grinding and cracking affect the white pixels?
L4	L_Hardness	Hardness of the grinding cloth for the L-axis of the grinding machine	quantitative	Whether the hardness of grinding and cracking is compared with the shadow warning of white pixels
L5	L_Pore Rate	The ratio of openings in the grinding cloth on the L-axis of the grinding machine	quantitative	Whether the open pore ratio has any effect on white pixels during grinding and cracking
L6	LPore Size	The open pore diameter of the L-axis grinding cloth of the grinding machine	quantitative	Whether the open pore size during grinding has any effect on white pixels
L7	LThickness	Thickness of the grinding cloth for the L-axis of the grinding machine	quantitative	Grinding thickness has no effect on white pixels
L8	L_SLURRY_FLOW	Ltable grinding with slurry flow	quantitative	The size of the slurry flow during grinding, and whether there is any shadow of the white pixels
L9	L_CHILLER_TEMP	Ltable grinding cooler temperature	quantitative	Effect of cooling effect on white pixels during grinding
L10	L_CHILLER_FLOW	Ltable grinding slurry flow	quantitative	Cooling effect during grinding, presence or absence of shadows on white pixels
L11	L_LOAD_AVE	Ltable grinding load	quantitative	Whether the amount of force loaded during grinding has any impact on white pixels
L12	Z_T_TORQUE_AVE	Ltable Table Torque	quantitative	Whether the amount of force loaded during grinding has any impact on white pixels
L13	L_HTORQUE_AVE	Ltable Head Torque	quantitative	Whether the amount of force loaded during grinding has any impact on white pixels
L14	LT_TEMP_AVE	Ltable Table Temperature	quantitative	Effect of the cooling effect on white pixels during grinding
L15	L_SLURRY_TEMP	Table Slurry temperature for grinding	quantitative	The impact of the slurry temperature on white pixels during grinding
R1	R_Compressibility	Compression rate of the grinding cloth on the R-axis of the grinding machine	quantitative	The impact of the compression ratio on white pixels during grinding and cracking
R2	R_Density	The space of the grinding cloth on the R-axis of the grinding machine	quantitative	The effect of density on white pixels during grinding and cracking
R3	R_Elasticity	Elastic modulus of the grinding cloth for the R-axis of the grinding machine	quantitative	The influence of elastic modulus on white pixels during grinding and cracking
R4	R_Hardness	The hardness of the grinding cloth for the R-axis of the grinder	quantitative	The effect of hardness on white pixels during grinding and cracking
R5	R_Pore Rate	Ratio of open pores on the grinding cloth on the R-axis of the grinder	quantitative	The effect of hole ratio on white pixels during grinding and cracking
R6	R_Pore Size	Open pore diameter of the R-axis grinding cloth of the grinder	quantitative	The impact of hole size on white pixels during grinding and cracking
R7	R_Thickness.I	Thickness of the grinding cloth for the R-axis of the grinding machine	quantitative	Effect of grinding thickness on white pixels
R8	R_SLURRY_FLOW	tabEr Grinding with sLurry flow	quantitative	The size of the slurry flow during grinding and whether it has any impact on white pixels
R9	R_CHILLER_TEMP	RtabRe grinding cooler temperature	quantitative	Cooling effect during grinding, impact on white pixels
R10	R_CHILLER_FLOW	tabEr grinding with sLurry flow	quantitative	Cooling effect during grinding, impact on white pixels
R11	R_LOAD_AVE	RtabRe Grinding load	quantitative	Whether the amount of force loaded during grinding has any impact on white pixels
R12	RT_TORQUE_AVE	RtabRe TabRe Torque	quantitative	Whether the amount of force loaded during grinding has any impact on white pixels
R13	R_H_TORQUE_AVE	RtabRe Head Torque	quantitative	Whether the amount of force loaded during grinding has any impact on white pixels
R14	RTTEMP_AVE	RtabRe TabRe temperature	quantitative	Cooling effect during grinding, impact on white pixels
R15	RSLURRY_TEMP	RtabRe For grinding sRurry temperature	quantitative	The impact of slurry temperature on white pixels during grinding
C1	C_Compressibility	Compression rate of the grinding machine’s C-axis grinding cloth	quantitative	The impact of compression ratio on white pixels during grinding and cracking
C2	C_Elasticity	Elastic modulus of the C-axis grinding cloth of the grinding machine	quantitative	The influence of elastic modulus on white pixels during grinding and cracking
C3	C_Hardness	Hardness of the C-axis grinding cloth of the grinding machine	quantitative	The effect of hardness on white pixels during grinding and cracking
C4	C_Pore Size	Open pore diameter of the grinding machine’s C-axis grinding cloth	quantitative	The impact of open pore size on white pixels during grinding and cracking
C5	C_Thickness	Thickness of the C-axis grinding cloth of the grinding machine	quantitative	Effect of grinding thickness on white pixels
C6	C_SLURRY_FLOW	CtabCe grinding with sCucCy flow	quantitative	The flow rate of sCuCCy during grinding and whether it affects white pixels
C7	C_CHILLER_TEMP 1	CtabCe grinding cooler tempeCatuCe	quantitative	Cooling effect during grinding, impact on white pixels
C8	CCHILLER_FLOW	CtabCe grinding with sCuCCy flow rate	quantitative	Effect of cooling effect on white pixels during grinding
C9	C_LOAD_AVE	CtabCe Grinding load	quantitative	Whether the amount of force loaded during grinding has any impact on white pixels
C10	CILTORQUEAVE	CtabCe TabCeTorque	quantitative	Whether the amount of force loaded during grinding has any impact on white pixels
C11	CHTORQUEAVE	C tabCe Head torque	quantitative	Whether the amount of force loaded during grinding has any impact on white pixels
C12	CTLTEMP_AVE	C tabCe TabCe tempeCatuCe	quantitative	Cooling effect during grinding, impact on white pixels
C13	C_SLURRY_TEMP	CtabCe sCuCCy tempeCatuCe for grinding	quantitative	The impact of sCuCCy temperature on white pixels during grinding

**Table 4:** White pixel severity classification of CIS product.

Defective White Pixels	Description	Classification
˂10%	Acceptable to customers	OK
≥10%	Not acceptable to the customer	NG

**Table 5:** Wafer fabrication factory processing conditions recommendation.

**Dependent Variable**					
**Term**	**ID**	**Type**			
White pixel ratio	Y1	qualitative			
**Independent Variable**					
**Term**	**ID**	**Type**	**Term**	**ID**	**Type**
L__Compressibility	L1	quantitative	R_CHILLER_FLOW	R10	quantitative
L_Density	L2	quantitative	R_LOAD_AVE	R11	quantitative
L_Elasticity	L3	quantitative	R_T_TORQUE_AVE	R12	quantitative
L_Hardness	L4	quantitative	R_H_TORQUE_AVE	R13	quantitative
L_Pore Rate	L5	quantitative	R_T_TEMP.AVE	R14	quantitative
L__Pore Size	L6	quantitative	R_SLURRY__TEMP	R15	quantitative
L_Thickness	L7	quantitative	C_Compressibility	C1	quantitative
L_SLURRY_FLOW	L8	quantitative	C_Elasticity	C2	quantitative
L_CHILLER_TEMP	L9	quantitative	C_Hardness	C3	quantitative
L_CHILLER_FLOW	L10	quantitative	C_Pore Size	C4	quantitative
L_LOAD__AVE	L.11	quantitative	C_Thickness	C5	quantitative
L_T_TORQUE._AVE	L12	quantitative	C_SLURRY_FLOW	C6	quantitative
L_H_TORQUE_AVE	L13	quantitative	C_CHILLER_TEMP	C7	quantitative
L__T_TEMP.AVE	L14	quantitative	C_CHILLER_FLOW	C8	quantitative
L_SLURRY_TEMP	L15	quantitative	C_LOAD_AVE	C9	quantitative
R_Compressibility	R1	quantitative	C_T_TORQUE._AVE	C10	quantitative
R_Density	R2	quantitative	C_H_TORQUE_AVE	C11	quantitative
R_Elasticity	R3	quantitative	C_T_TEMP.AVE	C12	quantitative
R_Hardness	R4	quantitative	C_SLURRY__TEMP	C13	quantitative
R_Pore Rate	R5	quantitative			
R_Pore Size	R6	quantitative			
R_Thickness	R7	quantitative			
R_SLURRY_FLOW	R8	quantitative			
R_CHILLER_TEMP	R9	quantitative			
C_SLURRY__TEMP	C13	quantitative			

**Table 6:** Significant factors in the A Zone.

A Zone				Significant Dependent Variable:	*p*
L axis	Estimate	Std. Error	z value	Pr (>|z|)	
L12	−6.897	2.938	−2.347	0.0189	*
L15	14.45	7.145	2.022	0.0431	*
R axis	Estimate	Std. Error	z value	Pr (>|z|)	
R8	−15.41	3.002	−5.133	2.85 × 10^−7^	***
R11	6.543	2.593	2.523	0.0116	*
C axis	Estimate	Std. Error	z value	Pr (>|z|)	
C12	−5.7434	1.493	−3.847	0.00012	***
C13	−3.577	1.2201	−2.932	0.00337	**

* Statistically significant at *p* < 0.05; ** statistically significant at *p* < 0.01; *** statistically significant at *p* < 0.001.

**Table 7:** Significant factors in the B Zone.

B Zone				Significant Dependent Variable:	*p*
L-axis	Estimate	Std. Error	z value	Pr (>|z|)	
L8	−2.4558	0.9179	−2.676	0.007461	**
L9	−2.3089	0.6803	−3.394	0.000689	***
L10	−3.0821	0.749	−4.115	0.0000387	***
L11	6.4189	0.7059	9.093	<2 × 10^−16^	***
L12	1.7802	0.6819	2.61	0.009043	**
L13	−2.4033	0.9709	−2.475	0.013315	*
L15	7.2222	1.3262	5.446	5.16 × 10^−8^	***
R-axis	Estimate	Std. Error	z value	Pr (>|z|)	
R3	−5.0515	2.1412	−2.359	0.018317	*
R4	60.3907	9.2698	6.515	7.28 × 10^−11^	***
R5	−19.6195	4.5493	−4.313	0.0000161	***
R6	−49.0227	8.2967	−5.909	3.45 × 10^−9^	***
R8	−3.1992	1.1355	−2.817	0.004841	**
R9	−4.8164	1.2629	−3.814	0.000137	***
R10	−2.0074	0.9057	−2.216	0.026664	*
R11	10.7062	1.003	10.675	<2 × 10^−16^	***
R12	−1.596	0.6528	−2.445	0.014491	*
R13	−2.1629	0.6962	−3.107	0.001892	**
R14	6.3412	1.2296	5.157	2.51 × 10^−7^	***
C-axis	Estimate	Std. Error	z value	Pr (>|z|)	
C2	6.0125	2.1692	2.772	0.00558	**
C3	2.9328	0.5219	5.619	1.92 × 10^−8^	***
C4	−6.2036	0.6342	−9.781	<2 × 10^−16^	***
C6	3.0909	1.0537	2.933	3.35 × 10^−3^	**
C7	2.729	0.8939	3.053	0.00227	**
C8	−2.5943	0.9874	−2.627	0.00861	**
C10	5.7189	0.794	7.202	5.92 × 10^−13^	***
C12	6.6029	1.4735	4.481	0.00000742	***
C13	−1.9878	0.705	−2.82	0.00481	**

* Statistically significant at *p* < 0.05; ** statistically significant at *p* < 0.01; *** statistically significant at *p* < 0.001.

**Table 8:** Test results of the ratio of training number to testing number.

Training/Testing	Testing Accuracy (%)			
	C50	CART	Random Forest	SVM
1/9	89.16%	81.19%	85.70%	84.37%
2/8	90.01%	86.29%	90.16%	87.78%
3/7	88.25%	86.88%	90.46%	89.44%
4/6	89.46%	92.05%	92.45%	90.66%
5/5	90.93%	92.36%	92.60%	91.17%
6/4	90.15%	91.04%	91.04%	90.15%
7/3	92.03%	91.24%	90.84%	88.84%
8/2	93.41%	93.41%	91.62%	91.02%
**9/1**	**93.98%**	**95.18%**	**95.18%**	**93.98%**
Mean	90.82%	89.96%	91.12%	89.71%
Std.	0.020	0.044	0.025	0.027

**Table 9:** Evaluation metrics of each model.

Model	Accuracy%	Precision%	Specificity%	F-Score
SVM	93.98	88.15	83.98	0.8350
C5.0	93.98	88.13	83.85	0.8319
RF	95.18	91.21	89.95	0.8978
CART	95.18	87.48	84.15	0.8330

**Table 10:** Experimental results of each model in the A Zone.

Method	C5.0		CART		RF		SVM	
	Training	Testing	Training	Testing	Training	Testing	Training	Testing
1	98.637	96.812	98.168	96.522	98.746	97.391	97.782	97.391
2	97.646	97.971	97.975	99.130	99.036	95.652	97.878	96.522
3	98.513	97.681	97.975	98.261	98.746	99.130	97.878	96.522
4	98.265	97.391	98.264	97.391	98.554	100.000	97.589	99.130
5	98.389	97.681	98.168	96.522	98.939	97.391	97.975	95.652
6	97.770	97.681	98.168	96.522	98.650	97.391	97.782	97.391
7	98.141	98.261	98.168	96.522	98.650	98.261	97.878	96.522
8	97.893	99.130	98.168	97.391	98.843	96.522	97.782	97.391
9	97.893	98.551	98.071	98.261	98.650	98.261	97.589	99.130
10	98.761	97.391	98.264	96.522	98.746	97.391	97.686	98.261
Max (%)	98.761	99.130	98.264	99.130	99.036	100.000	97.975	99.130
Min (%)	97.646	96.812	97.975	96.522	98.554	95.652	97.589	95.652
Mean (%)	98.191	97.855	98.139	97.304	98.756	97.739	97.782	97.391
Std.	0.384	0.658	0.102	0.957	0.147	1.243	0.129	1.159

**Table 11:** Experimental results of each model in the B Zone.

Method	C5.0		CART		RF		SVM	
	Training	Testing	Training	Testing	Training	Testing	Training	Testing
1	92.465	92.308	93.407	91.209	97.436	90.110	88.034	87.912
2	93.407	90.476	93.284	90.110	97.192	96.703	88.278	93.407
3	93.250	88.645	92.308	90.110	97.070	92.308	89.133	82.418
4	91.366	85.714	93.040	84.615	96.825	94.505	89.255	84.615
5	93.564	88.278	93.773	89.011	97.314	93.407	89.255	85.714
6	91.994	88.645	93.773	86.813	96.947	89.011	88.156	91.209
7	92.622	89.011	93.040	85.714	96.825	89.011	89.011	85.714
8	92.308	88.645	92.430	89.011	97.192	96.703	88.034	92.308
9	92.936	87.912	93.284	92.308	97.070	95.604	88.400	89.011
10	92.936	87.912	93.651	83.516	96.825	93.407	88.400	85.714
Max (%)	93.564	92.308	93.773	92.308	97.436	96.703	89.255	93.407
Min (%)	91.366	85.714	92.308	83.516	96.825	89.011	88.034	82.418
Mean (%)	92.685	88.755	93.199	88.242	97.070	93.077	88.596	87.802
Std.	0.679	1.719	0.512	2.933	0.216	2.933	0.509	3.606

**Table 12:** CART rules.

Rule	C10	L10	L11	L12	L13	R8	R12	R14	White Pixel or Not	CART Result
1			<0.46		≥0.12				0, N	129/138, 94%
2		<0.47	<0.46		<0.12				0, N	18/19, 95%
3			≥0.46	<0.81		≥0.48			0, N	24/30, 80%
4			≥0.46	<0.81		<0.48	≥0.84	<0.92	0, N	31/41, 76%
5	<0.084		≥0.46	<0.81		<0.48	<0.84		0, N	9/10, 90%
6		≥0.47	<0.46		<0.12				1, Y	25/27, 93%
7			≥0.46	≥0.81					1, Y	378/388, 97%
8			≥0.46	<0.81		<0.48	≥0.84	≥0.92	1, Y	16/19, 84%
9	≥0.084		≥0.46	<0.81		<0.48	<0.84		1, Y	210/238, 88%

**Table 13:** Factor ranking comparison between RF and CART.

Feature	Detailed Item	RF	CART
C10	C-AXIS TABLE TORQUE	4	5
L10	L-AXIS CHILLER FLOW	9	3
L11	L-AXIS LOAD	1	1
L12	L-AXIS TABLE TORQUE	3	2
L13	L-AXIS HEAD TORQUE	6	2
R8	R-AXIS SLURRY FLOW	5	3
R11	R-AXIS LOAD	2	-
R12	R-AXIS TABLE TORQUE	8	4
R14	R-AXIS TABLE TEMP	7	5

## Data Availability

Data are unavailable due to privacy and ethical restrictions. Partial data are available upon request.
